# New Delhi Metallo-β-Lactamase-Producing *Enterobacterales* Bacteria

**DOI:** 10.3201/eid2801.212106

**Published:** 2022-01

**Authors:** Yancheng Yao, Can Imirzalioglu, Linda Falgenhauer, SurvCARE Hesse Working Group, Trinad Chakraborty

**Affiliations:** Justus Liebig University German Center for Infection Research, Giessen, Germany.

**Keywords:** carbapenemase-producing bacteria, carbapenemase-producing Enterobacterales, Escherichia coli, Klebsiella pneumoniae, bacteria, antimicrobial resistance, New Delhi metallo-β-lactamase

**To the Editor:** Findlay et al. (*1*) reported a high prevalence (>25%) of New Delhi metallo-β-lactamase (NDM) among carbapenemase-producing *Enterobacterales* (CPE) in Switzerland during 2019–2020. To provide context, we analyzed recent trends for NDM-producers in Germany.

In a whole-genome-sequencing–based surveillance study conducted in 52 hospitals in Hesse, Germany, during 2017–2019, we detected 56 NDM-producing isolates among 346 CPE. The prevalence of NDM CPE increased from 6.3% (5/79) in 2017 to 15.0% (17/113) in 2018 and 22.1% (34/154) in 2019. A total of 56 alleles—28 NDM-1, 27 NDM-5, and 1 NDM-7—were detected in 31 *Klebsiella pneumoniae*, 18 *Escherichia coli*, and 3 *Citrobacter freundii* isolates and 1 isolate each of *C. portucalensis*, *Enterobacter hormaechei, K. grimontii*, and *K. variicola*. Isolates were derived from rectal swab (30), urine (11), respiratory tract (6), and other (9) specimens. More than 82% (23/28) of NDM-1 were associated with *K. pneumoniae* and 59% (16/27) of NDM-5 with *E. coli*; 41% of the NDM-CPE harbored a 16S-rRNA-methylase gene.

Among carbapenemase-producing *K. pneumoniae* isolates, NDM increased from 6.4% (3/47) in 2017 to 24.5% (13/53) in 2018 and 31.9% (15/47) in 2019; ≈50% (15/31) were sequence type 147. Thus, reports for Switzerland (*1*) and Italy (*2*) indicate that sequence type 147 NDM-carrying *K. pneumoniae* is a CPE clone rapidly emerging in Europe.

NDM accounted for 20% (2/10) of carbapenemase-producing *E. coli* in 2017, 11.1% (2/18) in 2018, and 24.1% (14/58) in 2019; the increase in NDM-5–producing *E. coli* was primarily associated with IncF-type plasmids (*3*). A subgroup harboring penicillin binding protein 3 modifications exhibited increased aztreonam/avibactam MICs of 2–8 μg/mL. Higher MICs (4–16 μg/mL) were associated with the presence of an additional highly conserved *bla*_CMY-42_–encoding IncIγ plasmid (*4*). We reiterate the call by Findlay et al. to implement genome-based surveillance studies to identify emerging clonal lineages and commonly occurring plasmids among carbapenemase-producing bacteria. 
